# Editorial: Deciphering the regulatory role of transcription factors in cancer immune infiltration

**DOI:** 10.3389/fonc.2025.1563949

**Published:** 2025-02-07

**Authors:** Qian Yang, Jinfen Xiao, Yujia Lan

**Affiliations:** ^1^ Department of Urology, Samuel Oschin Comprehensive Cancer Institute, Cedars-Sinai Medical Center, Los Angeles, CA, United States; ^2^ Department of Computational Biomedicine, Samuel Oschin Comprehensive Cancer Institute, Cedars-Sinai Medical Center, Los Angeles, CA, United States; ^3^ University of Texas MD Anderson Cancer Center, Houston, TX, United States; ^4^ College of Bioinformatics Science and Technology, Harbin Medical University, Harbin, China

**Keywords:** transcription factors, cancer, immune infiltration, tumor micro environment (TME), immunotherapy

## Introduction

Transcription factors (TF) are proteins that bind to specific DNA sequences and regulate gene transcription, playing essential roles in various cellular processes, such as cell proliferation, differentiation, and immune responses (1). Tumor immune infiltration, the process by which immune cells infiltrate tumor tissues is a critical component of the tumor microenvironment (TME) significantly influencing cancer progression and therapeutic outcomes (2). Dysregulated TF activities can contribute to cancer malignancies by modulating the expression of immune-related genes, impacting immune cell recruitment, activation, and function (3) and thus reshape the anti-tumor immune response (3). Understanding the regulatory role of TFs in cancer immune infiltration offers promising avenues for the development of novel therapeutic strategies and biomarkers for cancer treatment.

Tumors can be classified as “hot” and “cold” based on the degree and type of immune cell infiltration, immune cell activity, and the heterogeneity of the tumor microenvironment (4). Some cancer types are frequently identified as “hot” tumors, such as bladder, head and neck, kidney, liver, melanoma, and non‐small cell lung cancers. In contrast, ovarian, prostate, and pancreatic cancers are often classified as “cold” tumors due to low immune infiltration and immune response (4). Immunotherapy shows clinical benefits in some cancer types. However, challenges such as low response rate, high recurrence, and metastasis risk remain significant hurdles, particularly in treating “cold” tumors (4). TFs, especially master transcript factors, play a crucial role in driving lineage plasticity in cancers and modulating the tumor microenvironment. Targeting TFs could be explored as a potential therapeutic strategy for combinational therapy in cancer treatment. Identifying immune-related TFs could also help transform “cold” tumors into “hot” tumors by reconstructing the TME.

For this Research Topic, we aimed to unravel the intricate interplay between TFs and immune cells in the context of cancer immune infiltration. We investigated the potential functionalities of TFs in cancer through various mechanisms, including direct transcriptional regulation of immune-related genes, crosstalk with signaling pathways involved in immune cell activation, and interactions with other cellular components of the tumor microenvironment. In summary, deciphering the regulatory role of TFs in cancer immune infiltration is a crucial step toward understanding the complexities of the tumor microenvironment. This knowledge holds significant potential for advancing the development of more effective immunotherapy strategies.

## NF-κB transcription factors in the TME

The tumor microenvironment is a multifaceted and dynamic ecosystem comprising not only tumor cells but also various non-cancerous cells, secreted ligands, and the extracellular matrix (5, 6). This intricate environment plays a pivotal role in tumor progression, metastasis, and therapeutic response. NF-κB transcription factors were known for their central roles in inflammation and innate immunity, garnering attention for their critical involvement in cancer development and progression, as well as the configuration of the TME (7). A systematic review by Cao et al. emphasizes the multifaceted role of the NF-κB signaling pathway in regulating various aspects of the TME, including immune modulation, stromal cell function, angiogenesis, invasion, and the link between inflammation and tumorigenesis. They also discussed how NF-κB transcription factors recruit immune cells and their complex roles in immune cell types, such as T, B, NK, and DC cells. Last but not least, the links among tumor progression, metabolism, and immune infiltration have been summarized in this review. A review by Kumar and Gupta also discussed the connection between epigenetic regulation of one NF-kB downstream gene, NKG2D, and NK cell-based immunotherapy in cancer.

These findings suggest the crucial functions of transcription factors in the TME, and targeting these kinds of TFs could be a novel method to increase immune infiltration and enhance the efficiency of immunotherapy.

## NRF-1 function in the melanoma TME

Previous studies have identified the integrin-associated protein, CD47, to be a critical myeloid lineage checkpoint, which is overexpressed in various types of cancer cells, especially in melanoma, and can inhibit innate immunity-mediated anti-tumor responses (8–11). In Makwana et al.’s original study, they demonstrate that upregulation of CD47 occurs at the mRNA level and chromatin accessibility at the CD47 promoter region also changed in melanoma. Real-time PCR confirmed elevated CD47 mRNA production across multiple transcript variants. Analysis using the MotifMap algorithm identified binding consensus sequences for Nuclear Respiratory Factor-1 (NRF-1) within the CD47 proximal promoter region. Chromatin Immunoprecipitation (ChIP) assays further showed NRF-1 binding at predicted sites, with differential affinities observed between malignant and normal cell types, contributing to increased CD47 mRNA and protein levels in cancer cells. Bioluminescence reporter assays defined the minimal CD47 promoter region and identified the number of NRF-1 binding sites essential for the efficient activation of CD47. These findings highlight the regulatory role of NRF-1 to CD47 expression in melanoma. NRF-1 transcription factor serves as a critical regulatory hub, uniquely capable of controlling genes involved in both mitochondrial biogenesis and the evasion of innate immunity in the TME.

## PGC1α reshapes the TME in diverse cancers

The peroxisome proliferator-activated receptor gamma coactivator 1-alpha (PGC1α) importantly acts as a tissue-specific transcription coactivator by interacting with a multitude of transcription factors and regulates metabolic processes in cancer cells (12). PGC1α can also reshape the TME and modulate the immune response by coordinating metabolic networks between cancer cells and immune cells. Inhibition of PGC1α facilitates overcome of immune-evasive and therapeutic resistance. Therefore, a comprehensive elucidation of PGC1α in the TME is necessary. In this Research Topic, Wang et al. systematically reviewed the role of PGC1α in cancer and discussed specific functions of PGC1α in several cancer types. The magnitude of PGC1α at the transcriptional regulation level suggests that many crucial transcription factors contribute to the reshaping of the TME in cancer, such as FOXO1 and MYC. They summarized the significance of PGC1α in the cancer TME, especially in immune cells. Targeting PGC1 modulates immune cell metabolism and in turn alters the immune landscape of tumors by reducing immune suppression and enhancing the efficacy of immunotherapies. These studies indicate that transcriptional regulation sculptures TME and transcription coactivators such as PGC1α, could reshape immune cells in the microenvironment of diverse cancers.

## References

[1] Latchman DS. Transcription factors: an overview. Int J Biochem Cell Biol. (1997) 29:1305–12. doi: 10.1016/S1357-2725(97)00085-X 9570129

[2] Anderson NM Simon MC. The tumor microenvironment. Curr Biol. (2020) 30:R921–5. doi: 10.1016/j.cub.2020.06.081 PMC8194051 32810447

[3] Melssen MM Sheybani ND Leick KM Slingluff CL Jr. Barriers to immune cell infiltration in tumors. J Immunother Cancer. (2023) 11. doi: 10.1136/jitc-2022-006401 PMC10124321 37072352

[4] Wang L Geng H Liu Y Liu L Chen Y Wu F. Hot and cold tumors: Immunological features and the therapeutic strategies. MedComm (2020). (2023) 4:e343. doi: 10.1002/mco2.v4.5 37638340 PMC10458686

[5] de Visser KE Joyce JA. The evolving tumor microenvironment: From cancer initiation to metastatic outgrowth. Cancer Cell. (2023) 41:374–403. doi: 10.1016/j.ccell.2023.02.016 36917948

[6] Lan Y Zhao E Zhang X Zhu X Wan L S. A. Prognostic impact of a lymphocyte activation-associated gene signature in GBM based on transcriptome analysis. PeerJ. (2021) 9:e12070. doi: 10.7717/peerj.12070 34527446 PMC8401751

[7] Hayden MS Ghosh S. NF-kappaB in immunobiology. Cell Res. (2011) 21:223–44. doi: 10.1038/cr.2011.13 PMC3193440 21243012

[8] Willingham SB Volkmer JP Gentles AJ Sahoo D Dalerba P Mitra SS. The CD47-signal regulatory protein alpha (SIRPa) interaction is a therapeutic target for human solid tumors. Proc Natl Acad Sci U.S.A. (2012) 109:6662–7. 10.1073/pnas.1121623109 PMC3340046 22451913

[9] Ngo M Han A Lakatos A Sahoo D Hachey SJ Weiskopf K. Antibody therapy targeting CD47 and CD271 effectively suppresses melanoma metastasis in patient-derived xenografts. Cell Rep. (2016) 16:1701–16. doi: 10.1016/j.celrep.2016.07.004 27477289

[10] Jaiswal S Jamieson CH Pang WW Park CY Chao MP Majeti R. CD47 is upregulated on circulating hematopoietic stem cells and leukemia cells to avoid phagocytosis. Cell. (2009) 138:271–85. doi: 10.1016/j.cell.2009.05.046 PMC2775564 19632178

[11] Majeti R Chao MP Alizadeh AA Pang WW Jaiswal S Gibbs KD Jr.. CD47 is an adverse prognostic factor and therapeutic antibody target on human acute myeloid leukemia stem cells. Cell. (2009) 138:286–99. doi: 10.1016/j.cell.2009.05.045 PMC2726837 19632179

[12] Tan Z Luo X Xiao L Tang M Bode AM Dong Z. The role of PGC1alpha in cancer metabolism and its therapeutic implications. Mol Cancer Ther. (2016) 15:774–82. doi: 10.1158/1535-7163.MCT-15-0621 27197257

